# Identifying Common Data Elements to Achieve Injury-related Health Equity Across the Lifespan: A Consensus-Driven Approach

**DOI:** 10.1089/heq.2023.0044

**Published:** 2024-04-03

**Authors:** Kelsey M. Conrick, Brianna Mills, Molly Fuentes, Janessa M. Graves, Christopher St. Vil, Monica S. Vavilala, Eileen M. Bulger, Saman Arbabi, Ali Rowhani-Rahbar, Megan Moore

**Affiliations:** ^1^ School of Social Work, University of Washington, Seattle, Washington, USA.; ^2^ Harborview Injury Prevention and Research Center, University of Washington, Seattle, Washington, USA.; ^3^ Department of Epidemiology, School of Public Health, University of Washington, Seattle, Washington, USA.; ^4^ Department of Rehabilitation, University of Washington and Seattle Children's Hospital, Seattle, Washington, USA.; ^5^ College of Nursing, Washington State University, Spokane, Washington, USA.; ^6^ School of Social Work, University at Buffalo, Buffalo, New York.; ^7^ Department of Anesthesiology and Pain Medicine, University of Washington, Seattle, Washington, USA.; ^8^ Department of Trauma Surgery, Harborview Medical Center, Seattle, Washington, USA.

**Keywords:** injury, health equity, trauma registry, common data elements, trauma systems

## Abstract

**Background:**

Limited availability and poor quality of data in medical records and trauma registries impede progress to achieve injury-related health equity across the lifespan.

**Methods:**

We used a Nominal Group Technique (NGT) in-person workgroup and a national web-based Delphi process to identify common data elements (CDE) that should be collected.

**Results:**

The 12 participants in the NGT workgroup and 23 participants in the national Delphi process identified 10 equity-related CDE and guiding lessons for research on collection of these data.

**Conclusions:**

These high-priority CDE define a detailed, equity-oriented approach to guide research to achieve injury-related health equity across the lifespan.

## Introduction

Injuries are the leading cause of death for individuals 1−44 years of age in the United States.^1^ Disparities across the injury care spectrum (i.e., occurrence, treatment, and outcomes) exist by age, race, place, insurance, gender, and socioeconomic status.^2^ Research establishing these disparities and evaluating the effectiveness of efforts to achieve health equity often depends on data from electronic medical records and trauma registries, which document patient demographics, care received, and outcomes.^3^ However, data needed to conduct this research and evaluation are often not available or overaggregated. For example, one gap in the injury disparities literature includes the use of binary race categories (e.g., white/non-white).^2^ This practice perpetuates the racist centering of whiteness and precludes researchers from detecting the effects of interventions on subgroups experiencing the worst injury disparities. Additionally, data on sexual orientation, gender identity, and language fluency, among others, are rarely available to researchers and practitioners.

Common data elements (CDE) refer to a minimum set of questions and answers, which are uniformly collected and presented across research studies. This approach can enable cross-study comparisons, facilitate data aggregation and meta-analyses, and improve data quality.^4^ Several fields have established CDE, including traumatic brain injury^5^ and epilepsy research.^6^ We sought to reach national consensus on the CDE researchers and community partners identify as needed to conduct research to achieve injury-related health equity.

## Methods

We used a two-stage process to achieve consensus on CDE needed to conduct research and intervention evaluation to achieve injury-related health equity. First, we conducted an in-person workgroup utilizing the Nominal Group Technique (NGT)^7^ to identify an initial set of CDE; then we achieved national consensus on CDE to prioritize for collection using a modified web-based Delphi survey. The University Institutional Review Board approved all procedures.

The in-person workgroup followed the NGT process outlined by Humphrey-Murto et al.^7^ Twelve academic and community partners were invited to participate. Academic participants included a range of years of experience and injury mechanisms/intents. Community partners represented stakeholders from populations most affected by injury disparities, including disability, Latin(x), and children's advocacy groups.

Workgroup participants were provided with background information on current data-related limitations in injury-related health equity research and the structure and details of CDE collected in other fields (e.g., traumatic brain injury). During the in-person meeting, participants discussed currently collected equity data elements and identified those that should be prioritized in a set of CDE. A research team member took detailed notes and compiled a comprehensive list of CDE suggested. A facilitator then led the group in a discussion of each CDE to collapse similar elements and ensure specificity in the CDE named. Participants were also tasked with identifying key stakeholders for the Delphi process to create, standardize, and implement CDE.

The notetaker and facilitator met with the research team to discuss the recommendations made by the workgroup participants and agree upon the presentation of these findings in the Delphi process.

To collect expert opinion nationally and achieve consensus on the research priorities defined by the NGT workgroup, we utilized a web-based modification of the Delphi method to facilitate group judgments while eliminating pressure from dominant individuals.^8,9^ We identified a group of experts with relevant experience for identifying CDE critical to achieving injury-related health equity. We first identified authors with multiple publications identified through a scoping review of injury-related health disparities research,^2^ selected representatives from injury centers and national associations, and invited stakeholders identified through the NGT process.

Forty-five experts were recruited from 23 states and a variety of research, university, and community-based organizations. Participants were contacted through email with the survey link and were given 2 weeks to respond to each round; Rounds 2 and 3 were sent to participants 3 and 4 weeks after the prior round, respectively. Responses for all rounds were collected and managed using Research Electronic Data Capture (REDCap) tools.^10^

*Round 1: Identification.* Ten CDE proposed by the NGT workgroup were presented for rating on importance from 1 (very important) to 5 (very unimportant) on a Likert scale. Participants were instructed to decide importance based on feasibility, potential impact on populations with highest rates of disparities, and priority for inclusion in widespread data collection. Finally, participants were asked to provide any additional CDE for consideration.

*Round 2: Ranking.* Participants rank-ordered the 10 CDE from Round 1 against each other and rated the 5 CDE proposed from open-ended responses in the first survey independently from each other on a Likert scale from 1 (very important) to 5 (very unimportant).

*Round 3: Consensus.* In Round 3, the CDE were presented as they were ranked in Round 2. Participants indicated whether they agreed with the ranking (yes/no) and provided commentary on any rating with which they disagreed.

## Results

### NGT workgroup

The members of the workgroup discussed methods to address weaknesses of current injury datasets that frequently lack measures of health equity. Specifically, they recommended all injury datasets (e.g., National Trauma Data Bank) to collect a minimum set of equity-focused CDE. They suggested a Delphi process to achieve national consensus on the most critical CDE, limited to 5−10 elements. Finally, they advised research should solidify the question phrasing and categories (e.g., new race and ethnicity categories) for each element and pilot test the process of data collection and abstraction of new variables. Participants identified concerns about patient privacy for the collection of sensitive data, such as sexual orientation. This group also suggested a list of 10 CDE for consensus voting in the national Delphi process and identified stakeholders to be included in this process.

### National Delphi process

Twenty-three participants contributed to at least one round of the Delphi process. The final ranked 10 CDE are presented in Table 1. Participants in the Delphi proposed an additional three elements that are not included in the final list because they were deemed more appropriate for qualitative study rather than quantified and collected universally: lived experience of being a person of color, quality of education, and indicator of cultural alignment.

**Table 1.** Common Data Elements Identified and Ranked by Participants for Priority Inclusion in Trauma Registries and Injury Research

Rank	Common data element	Source where proposed
1	Long-term follow-up after discharge (functional outcomes, resilience, psychosocial outcomes)	Delphi
2	Geographical location of injury	NGT
3	Income and family size	NGT
4	More discrete racial and ethnic categories and ability to identify with more than one race	NGT
5	Primary language and fluency for patient and family	NGT
6	Preinjury housing and employment status	NGT
7	Level of social support	NGT
8	Availability of resources (e.g., transportation, medical care)	NGT
9	Geographic location upon discharge	NGT
10	Sexual orientation	NGT

NGT, Nominal Group Technique.

## Discussion

Using a consensus-based approach that included researchers and community partners, we identified 10 CDE for collection in research studies. More information is needed to characterize the processes by which discrimination, racism, and socioeconomic barriers drive injury-related disparities. Research to identify the context of these disparities to date has been limited by a lack of data specificity, which forces inappropriate, often racist, aggregation of disparity types such as race (e.g., white/non-white binary) or aggregations of injury mechanisms and intents.^2^ These aggregations reinforce systemic racism by centering white as the referent group and limit the ability to identify nuances in injury disparities needed to inform interventions. For example, Asian and Pacific Islanders are often found to have the lowest rates of injury,^11^ but aggregating these groups may obscure which subgroups are at higher risk for injury.

Furthermore, many studies rely on administrative datasets, which commonly misclassify race and ethnicity^12–14^ and omit other disparity types such as housing status. Any efforts to increase specificity of equity-related data should also be balanced with a need to ensure patient privacy and confidentiality, especially for extremely sensitive data, such as sexual orientation.

To better capture data on disparities and address limitations in current research, our stakeholders recommend a standard set of CDE to be collected for all injury research studies and incorporated into trauma registries, including the National Trauma Data Bank. This incorporation can address current gaps in knowledge about the processes by which discrimination, racism, and socioeconomic barriers drive injury-related disparities. Stakeholders across all stages of the research agenda development process stressed the importance of collaborating with communities to ensure both the questions and process of collection are culturally resonant. For example, if education level is to be collected, the options should be applicable to all patients rather than only those educated in the American system.

Once these CDE are implemented into trauma registries, critical analysis will be required to evaluate how intersectional identities influence inequities in injuries.^15^ This assessment begins with specifying the reason for each measure's use and explicitly examining the relevant social, environmental, and structural factors.^16^ Funders should require these evaluations to be explicit and grounded in critical theory (e.g., Critical Race, Postcolonial, or Queer Theories).^17^ Furthermore, when planning for data analysis, at a minimum, researchers should consider how their methods may inadvertently reinforce social hierarchies. Tools, such as the Public Health Critical Race praxis, can aid in designing antiracist methods when studying racial disparities.^18^

The method of using both NGT workgroups and a web-based Delphi process balanced the flexibility to generate open-ended recommendations with the need for specific CDE. Although the process is designed to minimize the influence of group dynamics or single individuals, bias is always a possibility. We engaged research and community experts throughout the process of agenda development, but some experts we contacted were unable to contribute, and other experts may not have been contacted. Furthermore, more time to devote to each stage of the agenda-development process may have resulted in additional identification of priorities, and priorities change over time.

## Conclusions

The CDE identified in this study represent a preliminary next step to achieve injury-related health equity. The next steps for research include studies to identify the question phrasing/answer options and data collection process that is feasible to implement into hospital, outpatient, and research settings and that are culturally resonant for patients. The systematic collection of these CDE will promote rigorous research to identify injury disparities more accurately and evaluate efforts to achieve injury-related health equity.

## Abbreviations Used

CDE: common data elements
NGT: Nominal Group Technique
REDCap: Research Electronic Data Capture
